# Assessment of health-related quality of life in patients with heart failure: a cross-sectional study in Saudi Arabia

**DOI:** 10.1186/s12955-022-02040-7

**Published:** 2022-08-30

**Authors:** Muslet Alharbi, Fahad Alharbi, Abdullah AlTuwayjiri, Yaqoub Alharbi, Yazeed Alhofair, Ahmed Alanazi, Faisal AlJlajle, Rehana Khalil, Osama Al-Wutayd

**Affiliations:** 1 Department of Family and Community Medicine, College of Medicine, Qassim University, Buraydah, Saudi Arabia; 2 King Fahad Specialist Hospital, Buraydah, Saudi Arabia; 3 Medical Intern, College of Medicine, Qassim University, Buraydah, Saudi Arabia; 4 Department of Family and Community Medicine, Unaizah College of Medicine and Medical Sciences, Qassim University, Unaizah, Saudi Arabia

**Keywords:** Health-related quality of life, HRQoL, SF-36 health survey, Saudi Arabia, Heart failure

## Abstract

**Background:**

As a precarious clinical condition and a public health problem, heart failure (HF) is associated with a significant burden of morbidity, mortality, and health care costs. As almost all of the published research has been conducted in Western countries, there is a need for culturally relevant studies in Saudi Arabia. This is the first study to investigate health-related quality of life (HRQoL) and its associated factors among Saudi patients with HF in the Qassim region.

**Methods:**

A cross-sectional study was conducted at the only tertiary care hospital in the Qassim region of Saudi Arabia during the period from November 2020 to July 2021. The participants were interviewed face-to-face by trained interviewers using the standard validated 36-item Short-Form Health Survey (SF-36) questionnaire for HRQoL assessment. The data were analyzed using STATA version 16.

**Results:**

The participants included 246 HF patients whose mean (SD) age was 56.7 (10.9) years. A majority of the respondents (80%, n = 197) were male, and 49% (n = 121) had an education level of less than secondary school. The median scores were high for the domains of social functioning (100 points) and bodily pain (75 points) and low for role-physical functioning (25 points). In general, the median scores for the physical and mental component summaries were 58.1 and 63.7, respectively. Patients with an education level less than secondary school were more likely to have a low physical component summary score (aOR 3.00, 95% CI 1.46–6.17), while female patients were more likely to have a low mental component summary score (aOR 2.67, 95% CI 1.38–5.16).

**Conclusions:**

Health-related quality of life was found to be moderate among these HF patients. Periodic HRQoL assessment is recommended for HF patients to minimize their physical and psychological concerns, particularly for patients with low education levels and female patients.

## Introduction

Heart failure (HF) is a public health problem with implications for health-related quality of life (HRQoL) [1]. In Saudi Arabia, the increased prevalence of HF over the past decade has imposed an economic burden on the health care system and on society [2]. According to the Centers for Disease Control and Prevention (CDC) and the World Health Organization (WHO), heart disease is the leading cause of death among the Saudi population [3, 4]; WHO data published in 2019 reported 3365.5 disability-adjusted life years (DALYs) per 100,000 population due to ischemic heart disease [5]. The probability of dying from cardiovascular disease, cancer, diabetes, or chronic respiratory disease in Saudi Arabia between the ages of 30 and 70 years is 20.9% [6].

A national multicenter survey in Saudi Arabia found that the mean age of patients with acute heart failure (AHF) and chronic heart failure (CHF) was 57–60 years [7], which is 10 years younger than that in developed countries such as Japan [8], the USA [9, 10], and European countries [11]. The explanation for this early occurrence of heart failure among the Saudi population is multifactorial and includes a high prevalence of diabetes mellitus and hypertension, which are major risk factors for coronary artery disease and HF [7, 12]. A further contributory factor is the upsurge of obesity in Saudi Arabia due to the country’s socioeconomic transition and adoption of a high-calorie Western diet [13]; other factors include a high prevalence of smoking [14].

HF has a significant impact on patients’ HRQoL, as the progressive loss of physical autonomy and the subsequent psychological distress due to impairment of social interaction impose lifelong limitations on daily activities [15]. Studies of impaired HRQoL as a marker of HF report an association with sex, age, marital status, social support, left ventricular ejection fraction (LVEF), duration of HF, psychosocial status, and comorbidities [15–21]. In studies of HRQoL among HF patients in Brazil [19], the United States [22, 23], Europe [24], and Canada [25], the vast majority of the patients assigned greater importance to their quality of life than to survival. To our knowledge, only one such study has been conducted in the Kingdom of Saudi Arabia (in the country’s eastern region), which found low physical and mental component summary scores and an overall low quality of life [26]. Therefore, this is the first study in the central region of the KSA that aimed to investigate HRQoL among Saudi HF patients and to identify factors associated with poor HRQoL in these individuals. We hypothesized that HF patients would have a lower physical and mental quality of life.

## Subjects and methods

### Study design and setting

The cross-sectional study of HF patients was conducted at King Fahad Specialist Hospital (KFSH) in the Qassim region between November 2020 and July 2021. KFSH is the region’s largest hospital, with more than 430 beds, and is also the region’s only tertiary care center. KFSH specializes in care for cardiac and oncology patients referred by primary care physicians and other hospitals in the region for assessment and admission [27]. The hospital is located in Buraidah, the capital of the Qassim region [28], which is one of the 13 administrative regions and is located in the center of Saudi Arabia, with a population of approximately 1.4 million. Approximately half of the population is aged less than 24 years, and one-fifth of the population has a university education or higher [29]; the average family income in this region ranked third compared to other regions [30]. It has an area of approximately 70,000 square kilometers, which represents 3.1% of the total area of Saudi Arabia. Moreover, agriculture is the primary source of the economy, contributing 17% of the GDP of the region. Qassim is also known as the “food basket” of Saudi Arabia. Approximately 60% of jobs are provided by the public sector, while the remaining 40% of jobs are provided by the private sector in the form of industries [31].

### Questionnaire

To assess HRQoL, we used the RAND 36-Item Health Survey 1.0 Questionnaire in Arabic version [32] of the 36-item Short Form (SF-36) Health Survey [33], which is a validated, self-report questionnaire. The instrument comprises 36 items with two composite measures of physical and mental components that encompass the following eight domains [33, 34]: physical functioning (PF), role limitations due to physical functioning (RP), bodily pain (BP), general health perceptions (GH), vitality (VT), social functioning (SF), role limitations due to emotional health (RE), and general mental health (MH). Each item is scored from 0 to 100, representing the worst to best quality of life. The physical component summary (PCS) score is calculated as the aggregate of four domains: PF (10 items), RP (four items), BP (two items), and GH (five items). The mental component summary (MCS) score is calculated as the aggregate of RE (three items), VT (four items), SF (two items), and MH (five items). Additionally, one unscaled item compares the respondent’s current health status with that in the past year [33]. In their assessment of the psychometric properties of the SF-36, Brazier et al. [35] reported satisfactory to excellent reliability coefficients of Cronbach’s α, which were > 0.75 for all scales except the SF domain (α = 0.73). Additionally, a strong correlation with the Nottingham Health Profile confirmed the instrument’s construct validity. Coons et al. [32] translated the instrument into Arabic and reported satisfactory psychometric results (highest α = 0.94 for the PF domain; lowest α = 0.71 for the general health domain).

Participants were classified into four groups depending on their HRQoL scores: scores from 0 to < 25 points, scores from 25 to < 50 points, scores from 50 to 74.9 points, and scores from 75 to 100 points. When the results were analyzed, none of the participants had a score less than 25, and thus, we classified the participants into 3 groups. The patients with scores of 0 to < 50 points were categorized as having low HRQoL, those with scores of 50–74.9 points were considered to have moderate HRQoL, and those with scores of 75–100 points were considered to have high HRQoL [26, 35, 36].

### Data collection

Patients diagnosed with HF were selected following admission to KFSH and during scheduled visits for assessment. Patients with cancer or major psychotic disorders were excluded from the study. The study’s purpose and objectives were explained to eligible patients, and those who consented to participate were interviewed face-to-face by trained interviewers using the standard validated SF-36 questionnaire for HRQoL assessment. The study was approved by the Regional Qassim Ethics Committee (reference number 675170) and conformed to the ethics guidelines of the Declaration of Helsinki. Written informed consent was obtained from all participants in the study.

### Sample size estimation

The sample size was computed using OpenEpi statistical software based on a previously reported correlation coefficient (0.2) among patients with HF [26], assuming adequate power (80%) and an alpha of 0.05. The minimum required sample size was 194 patients. In total, we recruited 246 patients diagnosed with HF, with an inclusion rate of 93%.

### Statistical analysis

The data were analyzed using STATA version 17 and are presented as frequencies with percentages for categorical data and medians with interquartile ranges (IQRs) for continuous variables. The Shapiro–Wilk test was used to assess the normality of the distribution of continuous variables that were not normally distributed; therefore, the Mann–Whitney U test and Kruskal–Wallis test were used to compare 2 groups or more, respectively. Multiple logistic regression analysis was used to detect for an association between sociodemographic characteristics and low quality of life in the physical and mental component summaries [37], and adjusted odds ratios (aORs) and 95% confidence intervals (95% CIs) were reported. A *p* value ≤ 0.05 was considered statistically significant.

## Results

The mean age of the participants was 56.7 (10.9) years; the majority (80%, n = 197) were male, and almost half of the participants (49%, n = 121) had less than a secondary school education. Ninety-nine (40.2%) participants had low scores for the physical component summary, and 72 (29.3%) had low scores for the mental component summary of the HRQoL assessment (details in Table 1). The median scores for the physical and mental component summaries were 58.1 and 63.7, respectively.

**Table 1:** Sociodemographic characteristics of the participants according to the PCS and MCS

Characteristics	Total	Physical component summary (PCS)			Mental component summary (MCS)		
		< 50	50–74	> 75	< 50	50–74	≥ 75
n (%)	246	99 (40.2)	89 (36.2)	58 (23.6)	72 (29.3)	90 (36.6)	84 (34.2)
Age, mean (SD)	56.7 (10.9)	59.4 (10.3)	54.2 (10.4)	55.9 (11.7)	57.2 (10.8)	57.1 (11.9)	55.9 (9.8)
*Sex, n (%)*							
Male	197	74 (37.6)	73 (37.1)	50 (25.4)	49 (24.9)	71 (36)	77 (39.1)
Female	49	25 (51)	16 (32.7)	8 (16.3)	23 (46.9)	19 (38.8)	7 (14.3)
*Education level, n (%)*							
≥ University	62	15 (24.2)	28 (45.2)	19 (30.7)	20 (32.3)	20 (32.3)	22 (35.4)
Secondary school	63	21 (33.3)	22 (34.9)	20 (31.8)	18 (28.6)	23 (36.5)	22 (34.9)
< Secondary school	121	63 (52.1)	39 (32.2)	19 (15.7)	34 (28.1)	47 (38.8)	40 (33.1)

The median scores and IQRs for the eight domains assessed by the SF-36 are displayed in Table 2. Overall, the median scores were ≥ 50 for all domains except the role of physical functioning domain, with a median of 25 points for quality of life. Findings for the physical component summary indicated that the participants had a median of 55 points for the physical functioning domain, 25 points for the role of physical functioning domain, and 75 points for the bodily pain domain. Participants reported a median score of 62.5 for their general health.

**Table 2:** The median scores and IQRs of the participants for the SF-36 quality of life domains

Subscale	Median	IQR
General health	62.5	25
Physical functioning	55	55
Role physical functioning	25	100
Role emotional functioning	66.7	100
Social functioning	100	25
Body pain	75	50
Vitality	50	25
Mental health	66	32

The mental component summary results indicated that the quality of social functioning domain had a median score of 100 points. Additionally, patients reported a median score of 66 points for the mental health domain, 66.7 points for the emotional role domain and 50 points for the vitality domain.

Table 3 shows that male sex, being aged 56 years or younger and having a higher educational level were shown to be significantly associated with a higher median score on the physical component summary (*p* = 0.024, *p* = 0.004, and *p* < 0.001, respectively). Male subjects showed significantly higher median mental component summary scores (*p* < 0.001) than females.

**Table 3:** Sociodemographic characteristics associated with the physical and mental component summaries

Characteristics		Median (IQR)	*p* Value
*Physical component summary*			
Age	> 56 years	50 (32.8)	0.004^a^*
	≤ 56 years	56.3 (30)	
Sex	Male	56.3 (33.8)	0.024^a^*
	Female	49.4 (21.3)	
Education level	≥ University	57.8 (28.8)	< 0.001^b^*
	Secondary school	61.3 (31.3)	
	< Secondary school	48.8 (33.1)	
*Mental component summary*			
Age	> 56 years	62.5 (31.2)	0.456^a^
	≤ 56 years	67.1 (32.8)	
Sex	Male	68.3 (31.7)	< 0.001^a^*
	Female	50.8 (23.8)	
Education level	≥ University	68 (32.9)	0.798^b^
	Secondary school	65 (35.5)	
	< Secondary school	62.5 (31.8)	

^a^Mann–Whitney *U* test, ^b^Kruskal–Wallis test, *p value ≤ 0.05

Multiple logistic regression showed that the patients with less than a secondary school education were more likely to have a low physical component summary score (aOR 3.00, 95% CI 1.46–6.17, *p* = 0.003), while female patients were more likely to have a low mental component summary score (aOR 2.67, 95% CI 1.38–5.16, *p* = 0.004) (Table 4).

**Table 4:** Multivariable analysis of the factors associated with poor quality of life (physical and mental quality of life)

Characteristics	Physical component summary			Mental component summary		
	aOR	95% CI	*p* Value	aOR	95% CI	*p* Value
*Age, years*						
> 56 years	1.71	0.96–3.02	0.066	1.94	0.70–2.38	0.407
≤ 56 years	Reference			Reference		
*Sex*						
Female	1.81	0.92–3.55	0.082	2.67	1.38–5.16	0.004*
Male	Reference			Reference		
*Education level*						
< Secondary school	3.00	1.46–6.17	0.003*	0.79	0.38–1.62	0.518
Secondary school	1.81	0.81–4.06	0.151	1.02	0.46–2.26	0.954
≥ University	Reference			Reference		

*p Value ≤ 0.05

## Discussion

As heart failure is a multifactorial clinical syndrome, interpreting quality of life among HF patients is a complex task [38]. Most previous studies have characterized HRQoL as subjective because of the self-reported nature of the data, which are based on patients’ own perceptions of their condition [39–41]. However, Alla et al. [42] and Scott et al. [43] eventually agreed on a multidimensional account that encompasses the physical, psychological, and social dimensions of HRQoL, all of which are incorporated in the SF-36 health survey [33–35]. Building on that approach, the present study found higher scores across almost all of the SF-36 domains compared to previous studies in the KSA’s Eastern region [26], Ethiopia [44], and Spain [18], which reported low HRQoL across all domains of the SF-36. This might be explained by methodological differences, variations in the history of comorbidities, duration of heart failure, treatment compliance, and differences in health quality. In the present study, the lowest scores were observed for the role limitations due to physical functioning domain, which is consistent with a study conducted in Spain [18] that indicated HF patients experience physical symptoms that include fatigue, dyspnea, chest pain, edema, and sleep difficulties, adversely affecting their physical ability to perform daily life activities [17, 45–50]. The present study found that the highest scores on the SF-36 were for the social functioning and bodily pain domains, which is consistent with a study in Spain [18]. These findings suggest that HF patients may try to maintain a positive psychosocial attitude despite physical limitations in their daily activities, positively impacting their HRQoL [22]. For patients in Saudi Arabia, the availability of personal attendants and family members for religious and sociocultural reasons may also be an important contributory factor [26].

The median score for the mental component summary was higher than that for the physical component summary in the present study. However, both of these scores were higher than those reported by previous studies [18, 26] and a recent meta-analysis of 14 studies [51]. The present results also found that participants with less than a secondary school education had poor PCS scores, indicating better physical functioning among those with high education levels. This confirms earlier evidence that education level is positively correlated with knowledge and awareness of one’s own physical health [52]. This might be because less educated patients may have difficulties following medical instructions and procedures, which in turn results in poor quality of life, and higher education is associated with better general health knowledge and more positive lifestyle factors [53]. An association has been demonstrated between a low educational level of HF patients and various quality of life aspects, including lower levels of physical functioning, higher anxiety, and poorer general health scores [39]. Moreover, HF patients with low education levels were found to have a 50% increased risk of hospitalization compared to highly educated patients [54]. This finding suggests an additional need for interventions in the form of medical or psychological counseling by health care practitioners to improve the functional and physical status of HF patients with low education levels.

Interestingly, male patients in the present study received higher MCS scores than female patients, indicating better mental functioning in males. This aligns with earlier evidence [55–58] that, in this context, men’s psychosocial adjustment is better than that of women. This sex difference in psychosocial adjustment is attributed to the strong effect of psychosocial stressors among women due to altered functioning of the hypothalamic–pituitary–adrenal axis (HPA axis) and autonomic nervous system [59]. HRQoL assessment is crucial in heart failure management and the optimization of guideline-directed medical therapy or interventions that can improve patient HRQoL; therefore, we recommend yearly follow-up assessments of HF patients for extended periods to acquire prospective data on their progress and to identify prognostic factors for the rehabilitation process.

### Strengths and limitations

The present study is notable for its sufficiently large sample size representative of the population and its utilization of a questionnaire validated for the Arabic language, which was in the form of a face-to-face questionnaire given in the only tertiary care setting of the region. The study’s limitations include its dependence on the inherent constraint of the SF36 instrument in the form of subjective reporting by the participants, which may lead to over- or underreporting. Moreover, HRQoL scores can be affected by the time since the diagnosis of heart failure. Most likely, the joy of having survived is high after the incident and will influence the reported HRQoL level. Selection bias cannot be excluded, as some patients may receive treatment for their condition in the private sector or a hospital in another region. The interviews were conducted by trained interviewers, but there is a chance that the interviewer influenced the interviews. Some sociodemographic characteristics were not included in this study, including BMI, marital status, employment status, smoking history, annual income and ejection fraction, because the SF-36 questionnaire had 36 items; therefore, we preferred to limit additional questions since we assumed that the majority of the participants would be elderly and thus less likely to cooperate for a long questionnaire. Additionally, the length of HF and illness were not addressed in the current study, which might have made the cohort significantly heterogeneous; thus, a further prospective study is recommended.

## Conclusion

Health-related quality of life was found to be acceptable among these HF patients. Periodic HRQoL assessment is recommended for HF patients to minimize their physical and psychological concerns, particularly for patients with low education levels and female patients.

## Data Availability

The dataset in this study is available from the first author upon reasonable request.

## References

[1] Savarese G, Lund LH (2017). Global public health burden of heart failure. Card Fail Rev.

[2] Alghamdi A, Algarni E, Balkhi B, Altowaijri A, Alhossan A. Healthcare expenditures associated with heart failure in Saudi Arabia: a cost of illness study. In: Healthcare, vol. 9, no. 8. Multidisciplinary Digital Publishing Institute, p. 988. 2021. 10.3390/healthcare9080988 PMC8391138 34442125

[3] Global Health—Saudi Arabia. https://www.cdc.gov/globalhealth/countries/saudi_arabia/default.htm. Accessed 8 Nov 2021.

[4] Saudi Arabia—World Health Organization. https://www.who.int/nmh/countries/sau_en.pdf. Accessed 8 Nov 2021.

[5] Global health estimates: Leading causes of DALYs. World Health Organization. https://www.who.int/data/gho/data/themes/mortality-and-global-health-estimates/global-health-estimates-leading-causes-of-dalys. Accessed 10 Nov 2021.

[6] WHO. Noncommunicable diseases: mortality. https://www.who.int/data/gho/data/indicators/indicator-details/GHO/probability-of-dying-between-exact-ages-30-and-70-from-any-of-cardiovascular-disease-cancer-diabetes-or-chronic-respiratory-(-). Accessed 10 Nov 2021.

[7] AlHabib KF, Elasfar AA, AlBackr H, AlFaleh H, Hersi A, AlShaer F, Kashour T, AlNemer K, Hussein GA, Mimish L (2011). Design and preliminary results of the heart function assessment registry trial in Saudi Arabia (HEARTS) in patients with acute and chronic heart failure. Eur J Heart Fail.

[8] Sato N, Kajimoto K, Asai K, Mizuno M, Minami Y, Nagashima M, Murai K, Muanakata R, Yumino D, Meguro T, Kawana M, Investigators ATTEND (2010). Acute decompensated heart failure syndromes (ATTEND) registry. A prospective observational multicenter cohort study: rationale, design, and preliminary data. Am Heart J.

[9] Adams KF, Fonarow GC, Emermann CL (2005). Rationale, design and preliminary observations from the first 100,000 cases in the Acute Decompensated Heart Failure National registry (ADHERE). Am Heart J.

[10] Gheorghiade M, Abraham WT, Albert NM, Greenberg BH, O’Connor CM, She L, Stough WG, Yancy CW, Young JB, Fonarow GC, OPTIMIZE-HF Investigators and Coordinators. Systolic blood pressure at admission, clinical characteristics, and outcomes in patients hospitalized with acute heart failure. JAMA 2006;296(18):2217–26. 10.1001/jama.296.18.2217 17090768

[11] Nieminen MS, Brutsaert D, Dickstein K, Drexler H, Follath F, Harjola VP, Hochadel M, Komajda M, Lassus J, Lopez-Sendon JL, Ponikowski P, Tavazzi L (2006). EuroHeart Failure Survey II (EHFS II): a survey on hospitalized acute heart failure patients: description of population. Eur Heart J.

[12] Abdulaziz Al Dawish M, Alwin Robert A, Braham R, Abdallah Al Hayek A, Al Saeed A, Ahmed Ahmed R, Sulaiman Al Sabaan F (2016). Diabetes mellitus in Saudi Arabia: a review of the recent literature. Curr Diabetes Rev.

[13] Guy GW, Nunn AV, Thomas EL, Bell JD (2009). Obesity, diabetes and longevity in the Gulf: is there a Gulf Metabolic Syndrome?. Int J Diabetes Mellitus.

[14] Algabbani AM, Almubark R, Althumiri N, Alqahtani A, BinDhim N (2018). The prevalence of cigarette smoking in Saudi Arabia in 2018. Food Drug Regul Sci J.

[15] Salyer J, Flattery M, Lyon DE (2019). Heart failure symptom clusters and quality of life. Heart Lung.

[16] Van Jaarsveld CH, Sanderman R, Miedema I, Ranchor AV, Kempen GIJM (2001). Changes in health-related quality of life in older patients with acute myocardial infarction or congestive heart failure: a prospective study. J Am Geriatr Soc.

[17] Zambroski CH, Moser DK, Bhat G, Ziegler C (2005). Impact of symptom prevalence and symptom burden on quality of life in patients with heart failure. Eur J Cardiovasc Nurs.

[18] Rodríguez-Artalejo F, Guallar-Castillón P, Pascual CR, Otero CM, Montes AO, García AN, Conthe P, Chiva MO, Banegas JR, Herrera MC (2005). Health-related quality of life as a predictor of hospital readmission and death among patients with heart failure. Arch Intern Med.

[19] Pelegrino VM, Dantas RAS, Clark AM (2011). Health-related quality of life determinants in outpatients with heart failure. Rev Lat Am Enfermagem.

[20] Son YJ, Song Y, Nam S, Shin WY, Lee SJ, Jin DK (2012). Factors associated with health-related quality of life in elderly Korean patients with heart failure. J Cardiovasc Nurs.

[21] Chung ML, Moser DK, Lennie TA, Frazier SK (2013). Perceived social support predicted quality of life in patients with heart failure, but the effect is mediated by depressive symptoms. Qual Life Res.

[22] Heo S, Lennie TA, Okoli C, Moser DK (2009). Quality of life in patients with heart failure: ask the patients. Heart Lung.

[23] Sauser K, Spertus JA, Pierchala L, Davis E, Pang PS (2014). Quality of life assessment for acute heart failure patients from emergency department presentation through 30 days after discharge: a pilot study with the Kansas City Cardiomyopathy Questionnaire. J Cardiac Fail.

[24] Fotos NV, Giakoumidakis K, Kollia Z, Galanis P, Copanitsanou P, Pananoudaki E, Brokalaki H (2013). Health-related quality of life of patients with severe heart failure. A cross-sectional multicentre study. Scand J Caring Sci.

[25] Ducharme A, Doyon O, White M, Rouleau JL, Brophy JM (2005). Impact of care at a multidisciplinary congestive heart failure clinic: a randomized trial. CMAJ.

[26] AbuRuz ME, Alaloul F, Saifan A, Masa’Deh R, Abusalem S (2016). Quality of life for Saudi patients with heart failure: a cross-sectional correlational study. Global J Health Sci.

[27] Saudi Ministry of Health. Statistical yearbook 2017. https://www.moh.gov.sa/en/Ministry/Statistics/book/Documents/Statistical-Yearbook-1438-Appendix.pdf. Accessed 11 Nov 2021.

[28] General Authority for Statistics. Kingdom of Saudi Arabia. Qassim region. https://www.stats.gov.sa/en/13. Accessed 11 Nov 2021.

[29] Population in Al-Qaseem region by gender, age group, and nationality (Saudi/Non-Saudi). General Authority for Statistics. Kingdom of Saudi Arabia. https://www.stats.gov.sa/en/5725. Accessed 11 Nov 2021.

[30] Household Income and Expenditure Survey. General Authority for Statistics. Kingdom of Saudi Arabia. https://www.stats.gov.sa/ar/37. Accessed 11 Nov 2021.

[31] Buraidah City Profile. M Ministry of Municipal and Rural Affairs. United Nations Human Settlements Programme (UN-Habitat). ISBN: 978-603-8279-03-8. 2019. https://unhabitat.org/sites/default/files/2020/04/buraidah.pdf. Accessed 11 Nov 2021.

[32] Coons SJ, Alabdulmohsin SA, Draugalis JR, Hays RD (1998). Reliability of an Arabic version of the RAND-36 Health Survey and its equivalence to the US-English version. Med Care.

[33] Ware JE, Sherbourne CD (1992). The MOS 36-Item Short-Form Health Survey (SF-36): I. Conceptual framework and item selection author (s). Med Care.

[34] Gandek B, Sinclair SJ, Kosinski M, Ware JE (2004). Psychometric evaluation of the SF-36® health survey in medicare managed care. Health Care Financ Rev.

[35] Brazier JE, Harper R, Jones NM, O'cathain A, Thomas KJ, Usherwood T, Westlake L (1992). Validating the SF-36 health survey questionnaire: new outcome measure for primary care. BMJ.

[36] Lahoud R, Chongthammakun V, Wu Y, Hawwa N, Brennan DM, Cho L (2017). Comparing SF-36® scores versus biomarkers to predict mortality in primary cardiac prevention patients. Eur J Intern Med.

[37] Zurita-Cruz JN, Manuel-Apolinar L, Arellano-Flores ML, Gutierrez-Gonzalez A, Najera-Ahumada AG, Cisneros-González N (2018). Health and quality of life outcomes impairment of quality of life in type 2 diabetes mellitus: a cross-sectional study. Health Qual Life Outcomes.

[38] Roger VL (2013). Epidemiology of heart failure. Circ Res.

[39] Riedinger MS, Dracup KA, Brecht ML, Investigators SOLVD (2002). Quality of life in women with heart failure, normative groups, and patients with other chronic conditions. Am J Crit Care.

[40] Majani G, Pierobon A, Giardini A, Callegari S, Opasich C, Cobelli F, Tavazzi L (1999). Relationship between psychological profile and cardiological variables in chronic heart failure. The role of patient subjectivity. Eur Heart J.

[41] Sneed NV, Paul S, Michel Y, VanBakel A, Hendrix G (2001). Evaluation of 3 quality of life measurement tools in patients with chronic heart failure. Heart Lung.

[42] Alla F, Briançon S, Guillemin F, Juillière Y, Mertès PM, Villemot JP, Zannad F, Investigators EPICAL (2002). Self-rating of quality of life provides additional prognostic information in heart failure. Insights into the EPICAL study. Eur J Heart Fail.

[43] Scott LD (2000). Caregiving and care receiving among a technologically dependent heart failure population. Adv Nurs Sci.

[44] Tarekegn GE, Gezie LD, Birhan TY, Ewnetu F (2021). Health-related quality of life among heart failure patients attending an outpatient clinic in the University of Gondar Comprehensive Specialized Hospital Northwest, Ethiopia, 2020: using structural equation modeling approach. Patient Relat Outcome Meas.

[45] Grady KL, Jalowiec A, White-Williams C, Pifarre R, Kirklin JK, Bourge RC, Costanzo MR (1995). Predictors of quality of life in patients with advanced heart failure awaiting transplantation. J Heart Lung Transplant.

[46] Boyd KJ, Murray SA, Kendall M, Worth A, Benton TF, Clausen H (2004). Living with advanced heart failure: a prospective, of patients and their carers based study community. Eur J Heart Fail.

[47] Gorkin L, Norvell NK, Rosen RC, Charles E, Shumaker SA, McIntyre KM, Capone RJ, Kostis J, Niaura R, Woods P, Hosking J, Investigators SOLVD (1993). Assessment of quality of life as observed from the baseline data of the Studies of Left Ventricular Dysfunction (SOLVD) trial quality-of-life substudy. Am J Cardiol.

[48] Nordgren L, Sörensen S (2003). Symptoms experienced in the last six months of life in patients with end-stage heart failure. Eur J Cardiovasc Nurs.

[49] Lainscak M, Keber I (2003). Patient's view of heart failure: from the understanding to the quality of life. Eur J Cardiovasc Nurs.

[50] Schiff GD, Fung S, Speroff T, McNutt RA (2003). Decompensated heart failure: symptoms, patterns of onset, and contributing factors. Am J Med.

[51] Moradi M, Daneshi F, Behzadmehr R, Rafiemanesh H, Bouya S, Raeisi M (2020). Quality of life of chronic heart failure patients: a systematic review and meta-analysis. Heart Fail Rev.

[52] Alkatheri AM, Albekairy AM (2013). Does the patients’ educational level and previous counseling affect their medication knowledge?. Ann Thorac Med.

[53] Barbareschi G, Sanderman R, Leegte IL, Van Veldhuisen DJ, Jaarsma T (2011). Educational level and the quality of life of heart failure patients: a longitudinal study. J Cardiac Fail.

[54] Sui X, Gheorghiade M, Zannad F, Young JB, Ahmed A (2008). A propensity matched study of the association of education and outcomes in chronic heart failure. Int J Cardiol.

[55] Pilote L, Dasgupta K, Guru V, Humphries KH, McGrath J, Norris C (2007). A comprehensive view of sex-specific issues related to cardiovascular disease. CMAJ.

[56] Möller-Leimkühler AM (2008). Women with coronary artery disease and depression: a neglected risk group. World J Biol Psychiatry.

[57] Norris CM, Ghali WA, Galbraith PD, Graham MM, Jensen LA, Knudtson ML, Investigators APPROACH (2004). Women with coronary artery disease report worse health-related quality of life outcomes compared to men. Health Qual Life Outcomes.

[58] Kazemi-Saleh D, Pishgoo B, Farrokhi F, Fotros A, Assari S (2008). Sexual function and psychological status among males and females with ischemic heart disease. J Sex Med.

[59] Möller-Leimkühler AM (2010). Higher comorbidity of depression and cardiovascular disease in women: a biopsychosocial perspective. World J Biol Psychiatry.

